# Evaluation of Digital Technologies for Home‐Based Assessment in People With Amyotrophic Lateral Sclerosis

**DOI:** 10.1002/acn3.70429

**Published:** 2026-05-20

**Authors:** Arne Mueller, Vanessa Vallejo, Mónica Povedano Panadés, Orla Hardiman, Véronique Danel‐Brunaud, Senda Ajroud‐Driss, Philippe Couratier, Jeremy Shefner, Anna Gokey, Mike DiCesare, Erin Lennox, Jens Praestgaard, Lucie Brujin, Peggy Allred, Ram Miller

**Affiliations:** ^1^ Biomedical Research, Novartis Basel Switzerland; ^2^ Bellvitge University Hospital Barcelona Spain; ^3^ Trinity College Dublin Dublin Ireland; ^4^ Hôpital Roger Salengro Lille France; ^5^ Northwestern University Feinberg School of Medicine Chicago Illinois USA; ^6^ CHU de Limoges Limoges France; ^7^ Barrow Neurological Institute Phoenix Arizona USA; ^8^ Linus Health Boston Massachusetts USA; ^9^ ZEPHYRx Troy New York USA; ^10^ Biomedical Research, Novartis Cambridge Massachusetts USA; ^11^ Evidence Generation and Decision Sciences. Sanofi Cambridge Massachusetts USA; ^12^ Novartis Pharmaceuticals UK Ltd London UK; ^13^ Novartis Pharmaceuticals Corporation Cambridge Massachusetts USA

**Keywords:** ALS functional rating scale‐revised, ALSFRS‐R, amyotrophic lateral sclerosis, digital endpoints, home‐based, self‐administered

## Abstract

**Objective:**

Digital technologies hold promise for transforming healthcare by enhancing personalized treatments and offer valuable opportunities to improve patient care. Here, we evaluated several novel, self‐administered, home‐based, digital endpoints for their association with corresponding conventional standard clinical measures (primary) in people living with Amyotrophic Lateral Sclerosis (ALS).

**Methods:**

This was a longitudinal study in people with ALS who were followed up to 9 months. A total of 33 participants were enrolled in the study. At each of six visits, participants were evaluated with a battery of conventional standard measurements to determine ALS disease progression and quality of life. Between visits, participants performed weekly home‐based self‐assessments with digital health technologies (DHT) and self‐administered ALSFRS‐R. Cross‐sectional analysis of DHTs anchored to ALSFRS‐R and longitudinal analyses were performed and compared to standard clinical measures.

**Results:**

Of the 33 participants, 20 completed the study, and 13 discontinued before completing the planned 9‐month follow‐up mainly due to disease progression. The distribution of various digital metrics in home‐based assessments corresponded well with the sub‐scores of ALSFRS‐R in the cross‐sectional analyses, with the strongest construct validity for digital speaking rate. In the longitudinal analysis, a weak but significant trend in most metrics was observed, with the strongest trend in the duration of the Timed Up and Go (high variability between participants).

**Interpretation:**

The findings from this study provide insights into the potential of digital endpoints to evaluate people living with ALS with the goal of reducing the burden of study participation and improving the efficiency of ALS clinical trials.

## 1 Introduction

Amyotrophic Lateral Sclerosis (ALS) is a progressive neurological disease characterized by degeneration of upper and lower motor neurons in the brain and spinal cord [1], leading to muscle atrophy and affecting the ability to initiate and control voluntary muscle movements such as walking, talking, eating, and breathing [2, 3]. The ALS Functional Rating Scale‐Revised (ALSFRS‐R) is widely considered the standard for assessing disease progression in ALS [4]. It is the most commonly used scale to evaluate functional status in ALS, despite having certain limitations for clinical research purposes [5]. On the ALSFRS‐R, a higher score indicates greater physical function and less disability [6]. A drop of 1 point per month on ALSFRS‐R is generally considered a clinically meaningful reduction in function for people with ALS.

Remote digital monitoring has become an important tool in the management of chronic disease [7]. In people living with ALS, digital measures collected in an individual's home environment may provide more objective measurements and allow for more frequent data collection than in‐clinic evaluations. In addition, digital endpoints may be more sensitive to change than traditional clinical scales, thereby providing deeper insights into different functional domains affected by ALS. In the context of clinical trials, digital endpoints may also reduce the burden of participation for people with ALS who may have functional limitations that make travel to the clinical site difficult.

In the present study, we evaluated several novel, self‐administered, home‐based, digital endpoints for their association with the corresponding conventional standard clinical measures (Primary). We also tested the feasibility, participant acceptance, and adherence of selected novel digital endpoints that capture aspects of the functional domains in the ALSFRS‐R.

## 2 Methods

### 2.1 Participants

This was a longitudinal natural history study without treatment in people living with ALS who were followed for a period of up to 9 months. A total of 33 participants were enrolled in the study. The main eligibility criteria are provided below.

#### 2.1.1 Inclusion Criteria

The study included participants aged ≥ 18 years who were diagnosed with possible, probable, laboratory‐supported probable or definite ALS, either familial or sporadic, according to the World Federation of Neurology Revised El Escorial criteria [8] of either bulbar or limb onset. In the opinion of the investigator, participants had to be able to communicate well, and understand and comply with the requirements of the study; to ambulate short distances (at least 3 m) with or without walking aid; grasp a pen and to write with at least one hand (either dominant or non‐dominant). Further, participants were required to be non‐ventilator dependent (noninvasive positive pressure ventilation required for less than 23 h per day) at the screening visit. Based on baseline ALSFRS‐R scores (mean 35.9) and functional inclusion criteria (ability to ambulate and grasp a pen), participants were generally in mild to moderate stages of ALS.

#### 2.1.2 Exclusion Criteria

Participants were excluded from the study if they had, in the opinion of the investigator, active medical conditions that were clinically significant and could jeopardize the participant's safety if participating in the study or limit their participation in the study. These include the following: active dementia, neurologic diseases other than ALS, or psychiatric illness that would have limited their participation in the current study; signs or symptoms of a clinically significant systemic viral, bacterial, or fungal infection within 30 days prior to the screening visit; severe dysarthria, requiring the assistance of a communication device for intelligibility; enteral tube feeding dependent; history of drug or alcohol abuse within the 12 months prior to baseline. Alcohol abuse was defined as a history of, or current alcohol misuse/abuse, which itself was defined as “5 or more standard drinks on the same occasion on each of 5 or more days in the past 30 days.”

### 2.2 Study Design and Methodology

**FIGURE 1:**
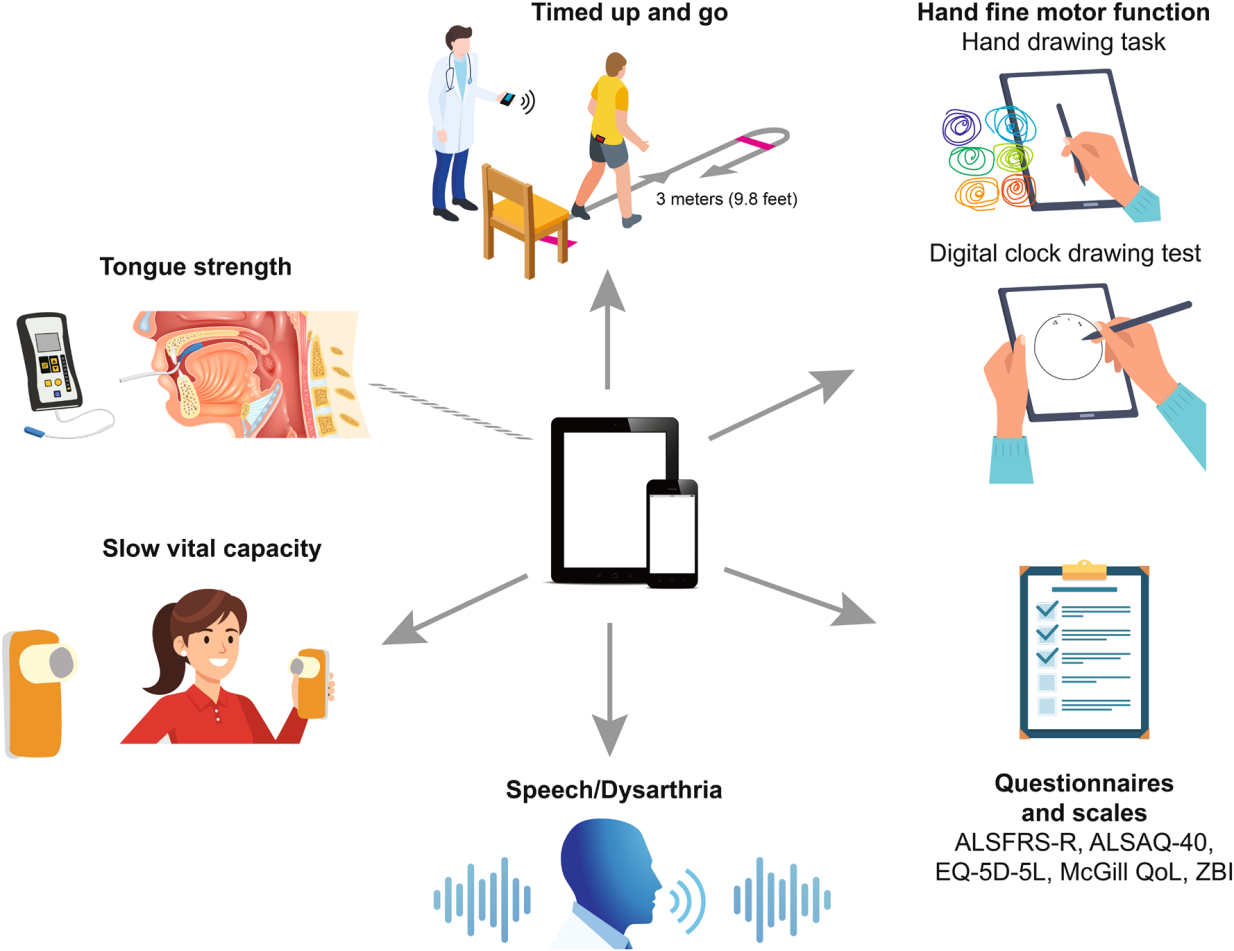
Digital setup. Clockwise: Instrumented Time Up and Go (TUG) with a Bluetooth‐connected sensor, spiral and clock drawing with a digital pen, questionnaires, speech recording and analysis with a built‐in microphone, slow vital capacity (SVC) with a Bluetooth‐connected hand‐held spirometer and tongue strength measurement. All assessments were scheduled and collected on a central application on an iPad, except for the tongue strength device that needs a USB connection to a computer. Slashed line indicates that this device is not connected to the iPad tablet.

The study was conducted in 5 centers in the USA and Europe, between 5 Oct 2021 and 15 December 2023. At the baseline visit, after full execution of the informed consent, each participant was evaluated with a battery of conventional standard measurements to measure ALS disease progression and quality of life: ALSFRS‐R [4, 6], EuroQoL five‐dimensions five‐levels (EQ‐5D‐5L) [9], Zarit caregiver burden interview (ZBI) [10], McGill quality of life scale (McGill QoL) [11], ALS assessment questionnaire −40 items (ALSAQ40) [12, 13, 14], slow vital capacity (SVC) [15], and timed up and go (TUG) [16]. Of these tools, only the ALSAQ‐40 (an ALS‐specific measure) has been specifically designed to assess QoL of people living with ALS. Details of digital endpoints and technologies are provided in Figure 1 and Supporting Information S1. Note, blood and urine biomarkers were collected three times during the study and may be published elsewhere.

The participants and their study partners were trained to use the digital assessment technologies and how to perform the home‐based digital assessment battery. Participants returned to the clinic 1 month after baseline and then approximately every 2 months for up to 5 in‐clinic follow‐up assessments over the 9‐month study. Throughout the study, participants were instructed to perform the digital assessment battery on a weekly basis at home. Sites conducted check‐ins with the participants and study partners by phone or video call about once a week or as needed to ensure that assessments were conducted as instructed. The digital battery included ALSFRS‐R (self‐reported version with simplified language; for the English version of the self‐administered ALSFRS‐R please see Table S1), an instrumented TUG test, speech assessment, fine motor skills (hand drawing tasks), spirometry (handheld), tongue strength, and cognitive assessments. These assessments were collected through an iPad tablet and a single study application with the assessment schedule, guiding the participant through the assessments (part of the Linus Health enterprise platform for researchers and healthcare providers). All but one device was connected via Bluetooth to the study application, and data from the connected devices were transferred directly to the study server (Figure 1). The tongue strength assessment was performed using the Iowa Oral Performance Instrument (IOPI Medical LLC, Carnation, WA, USA) device, which was not Bluetooth enabled. Although the iPad app guided the participants through the tongue strength (IOPI) test, data were only transferred via USB when participants returned with their at‐home device for the in‐clinic visits. Safety assessments were collected in the database of adverse events (AEs) and serious adverse events (SAEs) related to study conduct.

### 2.3 Statistical Analysis

For comparability of estimates in the statistical models the digital endpoints metrics were normalized to z‐scores within each parameter and across all participants and time points. Two main analyses were conducted: anchor‐based and longitudinal.

#### 2.3.1 Anchor‐Based Analysis

The anchor‐based analysis tested if digital endpoints distinguish between sub‐scores of ALSFRS‐R for a specific functional domain (e.g., if speaking rate derived from the speech assessments partitions with ALSFRS‐R bulbar function sub‐scores 1–4). Linear proportional odds models were fit to the ALSFRS‐R sub‐scores (1 to 4, the dependent variable in the model) by *z*‐score of the digital endpoint. The digital endpoint with the highest estimate was reported (the most extreme estimate of log odds ratio with *p* ≤ 0.01). Essentially, this model reported the log odds from moving to the next higher score when increasing the digital endpoint by one standard deviation unit. The model fits were used to derive a short list of endpoints for reporting; other endpoints may be meaningful as well, but are not reported here.

#### 2.3.2 Longitudinal Analysis

Due to disease progression in ALS, the clinical scales and digital endpoints were expected to change over time. This hypothesis was tested in a mixed linear effects model with the endpoint metric as the dependent variable (*Y*), study week as the independent variable (X), random intercept for participant and random slope (study week), i.e., this model assumes that participants had different baseline values and disease progression. The digital endpoint parameter with lowest *p*‐value for the study week fixed effect was reported. The same modeling approach was used for the clinical scales with the clinical scale metric as the dependent variable.

#### 2.3.3 Test‐Rest Reliability

This metric was expressed as the intraclass correlation (ICC) estimated from the longitudinal mixed effects models dividing the study week (random slope) variance by the total variance in the model fit (between and within participant variance).

#### 2.3.4 Coefficient of Variation

We implemented the definition of van Eijk et al. [17] to calculate the variation of an endpoint metric over time as the between participants variance of the study week random slope and the fixed effect study week estimate (i.e., inter‐participant study week variation over the grand slope of study week). The coefficient of variation (CV) was interpreted as a measure of generalizability of the endpoint over the participant population.

#### 2.3.5 Correlation

Pearson correlation was used if not otherwise indicated and the correlation coefficient was reported as *r*. For the exploratory purpose of this study, *p*‐values were used to filter the large number of endpoint metrics, and no correction for multiple testing was applied (no type I error correction).

#### 2.3.6 Adherence

Adherence was analyzed graphically with a heat map and logistic mixed effects regression. Each week an assessment was either performed (1) or not performed (0) by a participant. The digital assessment type was added as a fixed effect, with home‐based ALSFRS‐R adherence as contrast and the study week was added as a fixed numeric term as well as a random slope, and a random intercept was added per participant to account for different participant behavior.

### 2.4 Ethical Considerations

The study was conducted in compliance with the ethical principles of the Declaration of Helsinki. Informed consent was obtained from each participant in writing at screening before any study specific procedure was performed. The study was explained to a participant and their caregiver by the investigator or designee, who answered any questions, and written information was also provided. The study protocol, amendments, and informed consent documents were reviewed and approved by the responsible independent ethics committees or institutional review boards in each participating country/site prior to study initiation.

## 3 Results

Demographics and disease‐relevant metrics are summarized in Table 1. Of the 33 participants enrolled, 20 completed the study, and 13 participants discontinued prior to completion of the planned 9‐month follow‐up period, primarily due to disease progression. Data for discontinued participants was included up to the time of their discontinuation. The key questionnaire results are summarized in Table 2.

**TABLE 1:** Demographics.

Baseline characteristics	
Age in years, mean (SD)	61 (12)
Sex (one with NA)	
Male	20 (63%)
Female	12 (37%)
BMI, mean (SD)	27.7 (5.21) kg/m^2^
Disease duration at baseline, months, mean (SD)	30 (15)
Site of onset (number of participants)	
Upper limb	9
Lower limb	15
Bulbar	3
Trunk	6
Visits with ALSFRS‐R, mean (SD)	5 (1)
ALSFRS‐R – in‐clinic, mean (SD)	35.9/4.0
ALSFRS‐R progression points/month – in‐clinic, mean (SD)	−0.8 (0.1)
SVC at baseline – home‐based, % predicted, mean (SD)	83 (23)
Genetic carriers	
C9orf72	3
SOD1	2

Abbreviations: ALSFRS‐R, Amyotrophic lateral sclerosis functional rating scale‐revised; BMI, Body Mass Index; SD, standard deviation; SVC, slow vital capacity.

**TABLE 2:** Standard clinical assessments in longitudinal models.

Assessment	Intercept for study week	Slope for study week	*p*	Coefficient of variation	Intraclass correlation
ALSFRS‐R clinic	35.94	−0.19	< 0.0001	0.75	0.75
ALSFRS‐R home	37.77	−0.19	0.004	1.31	0.91
COA/EQ‐5D	67.89	−0.47	0.002	0.81	0.43
COA/Zarit	19.81	0.18	0.054	1.74	0.77
COA/McGill	87.10	−0.18	0.038	1.24	0.71
COA/ALSAQ40	128.95	1.44	0.011	1.33	0.85

*Note:* The models were fit to unscaled original data. Coefficient of variation (CV) indicates the overall variability between participants for this measure compared to the overall trend (across all participants). The intraclass correlation (ICC) is a measure of technical variability between measurements. A high ICC indicates test–retest reliability, while a high CV indicates heterogeneity between participants. Compare CV and ICC. Amyotrophic lateral sclerosis functional rating scale‐revised (ALSFRS‐R) home/clinic slopes (weekly progression rates) are the same, but the home‐version has higher ICC and varies more between participants (CV).

### 3.1 ALSFRS‐R Home and Clinic Comparison

The total score of the ALSFRS‐R self‐administered at home and the clinician administered ALSFRS‐R during clinical visits was compared by matching questionnaires (from home/clinic) of the same study week (Figure S1). From a linear model, the estimated overall difference between home and clinical total score was 2.26 points (model intercept) and with a slope of 0.89. This showed that the two methods were comparable, although scores from the self‐assessment at home were slightly higher than their matched in‐clinic scores. This is in broad agreement with previous home vs. in‐clinic comparisons of ALSFRS‐R [18].

### 3.2 Validation of Home and In‐Clinic Assessments (TUG and SVC)

SVC and TUG were conducted at home (self‐assessed) and in‐clinic. For SVC, the home‐based handheld device was used whereas in the clinic, the SVC was conducted in a pulmonary laboratory with standard local equipment. The at‐home assessments during the week of the clinical visits were correlated with the in‐clinic tests.

TUG in clinic was timed manually with a stopwatch by site staff in parallel to the sensor and app (automatically). The in‐clinic stopwatch timing was correlated with the sensor and app‐derived timing.

The correlation between the home and in‐clinic SVC was 0.88 (*n* = 76) and 0.99 for TUG (*n* = 37), though only 16 participants conducted the TUG in‐clinic, which may bias the correlation.

### 3.3 Anchor‐Based Analysis

**FIGURE 2:**
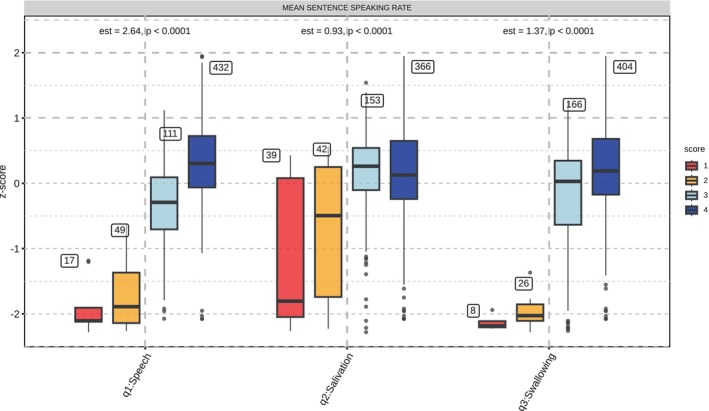
Digital speech metric partitioned by the ALSFRS‐R bulbar domain. The digital speech metric with the highest absolute regression coefficient (est) is shown including *p*‐values (*p*) and number of samples (speech sessions). Values are normalized to z‐scores. *Note:* This analysis tests construct validity against each question of the home‐based Amyotrophic Lateral Sclerosis functional rating scale‐revised (ALSFRS‐R) sub‐domains. A participant is measured several times in this study, and ALSFRS‐R sub‐scores are expected to change as the disease progresses. The real‐valued digital endpoint metric has a distribution for a specific score of an ALSFRS‐R question, and this distribution is thought to be shifted for the next higher and lower score (this is tested with the proportional odds statistical model). Scores of 0 are ignored as they indicate the absence of this specific function. The home‐based self‐administered ALSFRS‐R was used as the anchor instead of the standard in‐clinic questionnaire because it can be matched in time to the weekly digital endpoint sessions. The coefficient is the estimated effect size and is comparable between the different digital endpoints since input values are normalized (z‐scores).

The anchor‐based analysis for the digital endpoint statistics per functional domain (respiratory, bulbar, fine motor, gross motor) is summarized in Table 3 (all coefficients have a *p* < 0.0001). The anchor‐based analysis was visualized for the speech parameter “speaking rate” (syllables per second) as an example and presented in Figure 2. The proportional odds regression models a monotonic trend of the digital endpoint value through the 4 ordered scores, though the trend does not have to be linear. The mean sentence speaking rate had the strongest effect in these analyses compared to the other digital endpoints and functional domains.

**TABLE 3:** Digital assessments in anchor‐based analysis with ALSFRS‐R sub‐scores.

Domain	Digital endpoint metric	Question	Coefficient
Resp/speech	Minimal monotonicity of speech	q11: Dyspnea	−1.00
Bulbar/speech	Mean sentence speaking rate	q1: Speech	2.64
Bulbar/tongue	Maximum tongue pressure	q2: Salivation	1.71
Fine/clock	Simple composite motor function score	q4: Handwriting	0.67
Fine/spirals	Mean pen pressure (trace, dominant hand)	q5: Cutting food and handling	1.40
Gross/TUG	Total duration	q9: Walking	−0.87
Resp/SVC	Percent predicted volume	q13: Respiratory insufficiency	1.69

*Note:* ALSFRS‐R domains Resp/respiratory (questions 11–13), Bulbar (questions 1–3), Fine/fine motor function (questions 4–7), Gross/gross motor function (questions 8–10). The coefficient is from the proportional log odds ratio model for the question scores and the digital endpoint metric z‐score (all *p*‐values are < 0.0001 and are not displayed).

### 3.4 Comparison to Clinical Standard Quality of Life Measures

Scores for McGill QoL, ZBI, EQ‐5D‐5L VAS and ALSAQ40 were correlated with all digital endpoints. In summary: The weekly at‐home collected digital endpoints were mapped to the closest completed questionnaires in time with a maximum gap of 4 weeks. The correlations were significant but not strong: TUG turn duration increases with worse feelings in the McGill QoL (*r* < −0.54, *p* < 0.002); burden as assessed by ZBI increased with decreasing pen tip pressure (free/dominant hand) from the spiral drawing (*r* = −0.41, *p* < 0.0001) and maximum tongue pressure increases with increasing EQ‐5D‐5L quality of life score (*r* = 0.40, *p* < 0.004). The ALSAQ40 sub‐scores per category were similarly correlated to the digital endpoints. Notably, the mean sentence articulation precision was strongly negatively correlated with the combined score for eating/drinking and communication (ALSAQ‐40 questions 3 and 4, *r* = −0.77, *p* < 0.0001). The sub‐score for mobility was correlated with the turn duration of the TUG (*r* = 0.70, *p* ≤ 0.05) and the sub‐score for “activities” (question 2) was negatively correlated with top pen tip pressure of the spiral drawing task (free/dominant hand, *r* = −0.62, *p* < 0.0001).

The study did not recruit enough participants with confirmed C9ORF72 mutation (*n* = 3, aim was 10) to assess potential cognitive impairment, and we therefore did not analyze the cognitive assessment battery.

### 3.5 Longitudinal Analysis of Digital Endpoints and Comparison to ALSFRS‐R

Longitudinal analysis is summarized in Table 4. These were the digital endpoint metrics with the most significant trends over time independent of the ALSFRS‐R. The statistical parameters definitions are the same as in Table 2. The trends over time as estimated by the slope are weak for all endpoints. This is mainly related to variability between participants as indicated by the rather high CV and may reflect the heterogeneity of the disease symptoms. Table 5 shows the longitudinal statistics per ALSFRS‐R functional domain (home and in‐clinic). The scores were normalized to z‐scores per domain to allow comparison of the slopes from the digital endpoints statistical models in Table 4. Some ALSFRS‐R sub‐scores had a stronger trend over time compared to the digital metrics, but others were comparable or stronger for the digital endpoints (compare Tables 4 and 5).

**TABLE 4:** Longitudinal analysis of digitial assessments.

Domain	Key	Slope	*p*‐value	Coefficient of variation	Intraclass correlation
Bulbar/speech	Mean speaking rate	−0.0083	0.0097	1.72	0.96
Bulbar/tongue	Max tongue pressure	−0.0076	0.2280	3.08	0.90
Fine/clock	Percent ink time	0.0144	0.0010	1.06	0.62
Fine/spirals	Mean angular velocity (free, dominant hand)	0.0172	0.0011	1.32	0.70
Gross/TUG	Total duration	0.0235	0.0020	0.84	0.79
Resp/SVC	Percent predicted volume	−0.0166	0.0087	1.74	0.89

**TABLE 5:** Longitudinal statistics for ALSFRS‐R domains and total (normalized scores).

Domain	Slope	*p*‐value	Coefficient of variation	Intraclass correlation
Clinic				
Bulbar	−0.014	0.0051	1.446	0.824
Fine	−0.020	< 0.0001	0.740	0.863
Gross	−0.016	0.0002	0.826	0.891
Resp	−0.013	0.0148	1.263	0.473
Total	−0.025	< 0.0001	0.747	0.752
Home				
Bulbar	−0.017	0.0338	2.295	0.889
Fine	−0.021	< 0.0001	0.998	0.944
Gross	−0.019	< 0.0001	1.013	0.958
Resp	−0.015	0.0155	1.921	0.882
Total	−0.026	0.0038	1.306	0.909

*Note:* per domain the amyotrophic lateral sclerosis functional rating scale‐revised (ALSFRS‐R) sub‐score ranges from 0 to 12, and for simplicity and comparability ordinary linear mixed models were fit similar to that for the digital endpoint (rather than log‐odds ratio models). Scores were normalized for comparability with the models for digital metrics in Table 4.

**FIGURE 3:**
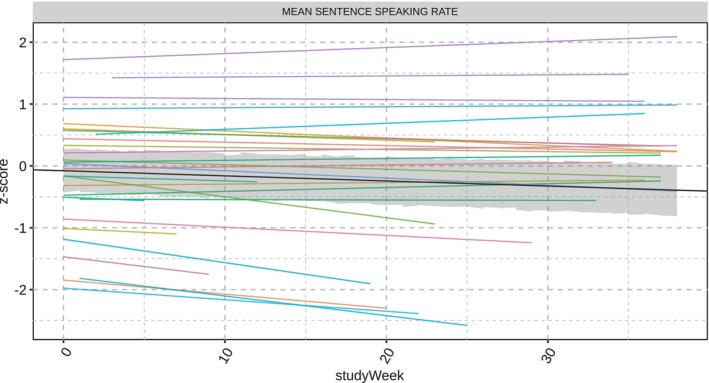
Modeled speaking rate over time in all participants (colored lines) and mean trend (wider black line).

**FIGURE 4:**
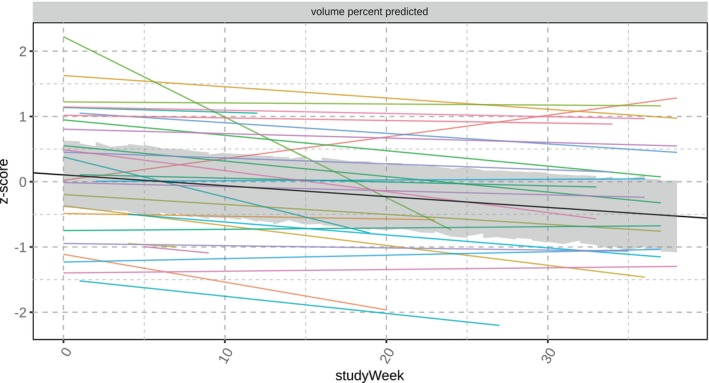
Modeled percent predicted volume for SVC for all participants (colored lines) and mean trend (wider black line).

Model statistics for the functional domains were comparable between home and in‐clinic ALSFRS‐R, though surprisingly the in‐clinic respiratory domain had lower ICC. The ALSFRS‐R functional domains pool different aspects such as speech, swallowing and salivation for bulbar function, whereas a digital endpoint often addresses one specific aspect of a function which may be reflected in the differences of the model statistics. As an example, Figure 3 shows the slopes per participant and the mean trend over time for mean speaking rate. Only a few participants in this study had bulbar onset and/or bulbar progression. In comparison, more participants showed a reduction in percent predicted lung volume over time (Figure 4), and the overall trend was stronger over time than for speaking rate.

**FIGURE 5:**
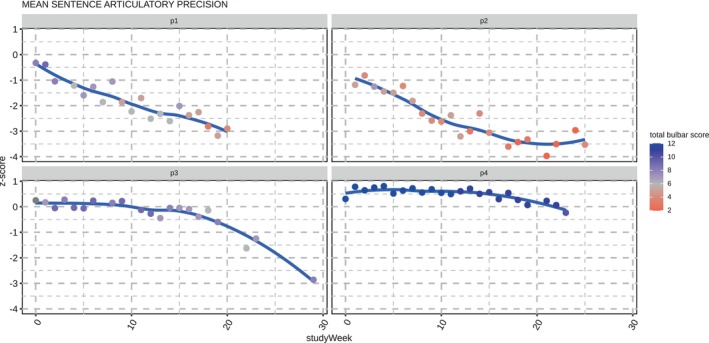
Digital speech profile for selected participants for articulation precision. *Note:* Each data point represents a home‐based speech assessment color coded by the home‐based ALSFRS‐R total bulbar score of the same week. The trend line is a polynomial fit.

Figure 5 shows the digital articulation precision for four selected participants. It exemplifies different trends and ranges. Participant p2 had bulbar onset and presented with low bulbar scores at the beginning of the study. A floor effect of the digital speech metric became evident at around week 20. Participant p1 shows linear decline, but the digital metric decline was faster than for the total bulbar score. Participant p3 had intermediate scores up to week 15, followed by a rapid decline after week 20. Again, the decline in the digital metric was stronger than for the bulbar score. Participant p4 was stable with high scores up to about week 12, and then showed slow decline; this was visible in both the ALSFRS‐R bulbar score and the digital metric.

### 3.6 Adherence

Adherence to the once‐a‐week home assessment schedule was assessed. The mean adherence was 0.58 with the lowest of 0.35 for tongue strength and the highest of 0.67 for the self‐administered ALSFRS‐R. A drop in adherence was observed over the duration of the study (see Figure S2 and Table S2). This is a known issue of DHTs in longitudinal studies [19]. Although there was an overall poor adherence for tongue strength measurement with IOPI, this adherence estimate was likely artificially low because data from participants who forgot to bring their home IOPI device to the clinic could not be extracted and uploaded at the site by clinical personnel (the IOPI device was not Bluetooth nor internet enabled). Consequently, the actual adherence to the weekly performance of the tongue strength measurement could not be accurately determined. During the study, TUG adherence decreased. Some participants reported they did not feel safe continuing to perform the measure at home due to disease progression. Though there were no falls associated with the TUG, this assessment was stopped for some participants upon their request or that of the site investigator secondary to safety concerns. TUG has highly significant log odds for non‐adherence (−0.96, *p* < 0.0001), but the overall drop in adherence over time across all assessments was small though significant (*p* < 0.001, log odds −0.07). On average, per month participants conducted most assessments more than 2 times.

### 3.7 Patient Acceptance

Participants completed a satisfaction questionnaire three times for each digital endpoint category (except for the home‐based self‐reported ALSFRS‐R) during the study at month 1, 5, and 9 (EOS). Figure S3 shows the reported level of satisfaction for each of the questions and assessment categories using a color code ranging from 0 (negative, gray shade) to 5 (positive, blue shade). Question 9 and 10 (“this is a meaningful assessment”, and “it helps controlling the disease”) received relatively poor scores for most of the assessment battery. There was a slight trend for some questions over time: “Easy to use” improved from the first to the second to the third questionnaire, but “got used to it” got a slightly poorer rating at the second and third times. Overall, there was a weak but significant trend towards negative rating over time (*p* < 0.01), suggesting that participants in this study became more disappointed. The questionnaire in Table S3 contains complete and understandable sentences.

### 3.8 Safety

In this study, there was one AE related to the study conduct. One participant experienced nausea due to the use of IOPI bulb. This event was reported as mild in severity and resolved. During the study, there were no SAEs related to study conduct. Four deaths were reported in this study, and all deaths were unrelated to the study conduct. Of the 4 deaths, 3 were due to ALS disease progression, and one death was due to stroke.

## 4 Discussion

The correlation between ALSFRS‐R scores obtained in‐clinic versus at home confirms that ALSFRS‐R can be effectively used for self‐reported assessments [6]. However, the field may benefit from harmonizing language and participant instructions for home‐based versions of the ALSFRS‐R [20]. Participants tend to rate themselves higher than clinicians.

In the cross‐sectional analyses, the distribution of various metrics in home‐based assessments corresponded well with the sub‐scores of the ALSFRS‐R. Particularly strong associations were found between digital metrics and ALSFRS‐R sub‐domains such as speech and tongue strength, which are associated with bulbar sub‐scores, as well as between SVC and respiratory function. However, weaker associations were observed for metrics such as TUG with gross motor function and drawing with fine motor function. The clinical meaning of digital fine motor function was difficult to interpret and may be prone to training effects.

In the longitudinal analysis, a weak but significant trend in all metrics was observed with the exception of tongue strength, and slopes within the same digital endpoints showed high variability between participants. For instance, when examining the three participants with bulbar onset, a significant decrease in digital speaking rate over time can be observed in each of them. Although not measured in this study, a recent publication indicates the listener effort speech endpoint may be a predictor of bulbar progression that is meaningful to patients and caregivers [21]. In this study, we included a similar speech endpoint articulatory precision [22, 23].

The study inclusion criteria may have resulted in the selection of a higher functioning, more slowly progressing population contributing to the weak trends in the longitudinal analysis. However, our cross‐sectional analysis showed different value distributions of several digital endpoint metrics within and between participants and irrespective of whether a participant's ALSFRS‐R score changes over time. This indicates a higher resolution of the digital endpoints compared to the ALSFRS‐R sub‐scores, which may also show stronger and possibly earlier detection of decline over time in participants with faster disease progression.

The individual digital measures were chosen based on their relationship to the functional domains of the ALSFRS‐R clinical score. The ALSFRS‐R total score is calculated as the sum of the 12 individual ALSFRS‐R questions. Similarly, it is possible that a composite score, derived from the individual digital measures, could holistically measure ALS progression. This hypothesis was supported by our findings that the ALSFRS‐R sub‐scores showed similar weak trends as the digital scores, yet the ALSFRS‐R total score had a stronger decline than any of its sub‐scores.

Some digital endpoints showed scale effects (flooring, ceiling), e.g., when a participant had low performance in a specific functional domain, further decline may not be detectable. However, our primary interest was to detect early, subtle changes, as these may be needed to prove effects of a therapeutic intervention. In our study, for some participants the digital endpoints detected the beginning of a functional decline earlier than the self‐reported ALSFRS‐R. Perhaps a composite digital score could detect early changes across a broad study population. Overall, considering a participant‐centered approach and both ALSFRS‐R scores as well as digital metrics may provide a more comprehensive understanding of disease progression in ALS.

Some assessments were less robust than others regarding conduct and data quality. Specifically, SVC and TUG were less tolerant of deviations. This may bias the results, as not all participants were able to perform the tests over the course of the study. This bias was evident in the adherence analysis. Conversely, we observed high correlation between the in‐clinic measurements for SVC and TUG and their home‐based assessments, showing that in principle these tests can be performed with similar accuracy as in‐clinic.

In terms of participant experience, although the perceived meaningfulness of the technology was judged poorly, the overall experience with the technology was reported as being good by the participants. Participant information and training were important to ensure understanding of the assessments and aspects of ALS the assessment was measuring. It is also important for the digital endpoints to capture the functions that are important to people with ALS and that change over time secondary to disease progression.

Adherence to the weekly schedule was good initially but decreased over time. Note, that our assessment battery was developed to measure the functional domains of the ALSFR‐R, however, these may not be directly meaningful for the participants and may have impacted adherence. We did not perform a down‐sampling analysis, but bi‐weekly or monthly assessments may provide similar clinical conclusions and are less burdensome for participants. Also, frequent assessments may introduce learning effects, and care must be taken to change the stimuli in the assessments such as using different sentences in the speech tests (in our study these sentences were changed several times).

Technology function during study conduct did not include offline assessment support, which led to data loss in cases where a participant did not have access to a network connection. Network connectivity issues including use of the international eSIM delayed data transfer which also led to data loss. These issues were resolved during the study which led to improvement and robustness of data collection.

SVC capture at home requires thorough training and frequent monitoring. Video coaching was available but should have been used more frequently to retrain and monitor participants. SVC data quality issues occurred in our study and many sessions had to be excluded. More frequent re‐training (via the integrated online training functionality of the app) may have increased the number of valid SVC sessions.

Several participants had decline in gross motor function secondary to disease progression which is reflected in the TUG results. From our study experience, we do not recommend at home TUG because of safety concerns, low acceptability and technical difficulties. Alternative tests should be explored such chair‐rise tests or continuous real‐world actigraphy [17].

## 5 Recommendations

The ALSFRS‐R can be reliably self‐administered at home. The speech endpoint is mature, has valid construct validity and tracks changes over time although not across all participants, and can be recommended for inclusion in clinical studies. Gross motor assessment is important, but more robust assessments should be developed, e.g., real‐world physical activity and mobility. The drawing tasks as hand function tests have low operational burden and show trends over time but are difficult to interpret in this study population and thus require further research for inclusion in ALS clinical studies. The participants' understanding of the assessments is important and should be highlighted as it may increase adherence and acceptance. Including the patient's voice in developing meaningful endpoints for the disease is strongly recommended. Participant perspectives are valuable to define treatment benefits and ensure clinical trials are more patient‐centric. The integration of the assessments into a single study application provided a single point of interaction with the participant, but the integration of multiple assessment modalities into a single platform should be further developed and made more robust. A minimal set of easy to derive and to interpret metrics from the digital endpoints across the relevant functional domains may be integrated into a composite disease progression score. Such score needs validation.

Our findings demonstrate that digital endpoints, particularly self‐administered ALSFRS‐R and speech assessments, can be reliably implemented in home‐based settings and offer valid, sensitive measures of ALS. We recommend the inclusion of validated digital endpoints in ALS clinical trials to enhance patient‐centricity, improve trial efficiency, and accelerate therapeutic development.

## Author Contributions

All authors contributed to the conception and design of the study, data analysis and interpretation of the data, drafting the manuscript or revising. All authors read and approved the manuscript for publication.

## Funding

This work was funded by Novartis Pharma AG.

## Conflicts of Interest

The authors declare no conflicts of interest.

## Supporting information


**Figure S1:** Comparison of self‐administered home and clinic administered ALFRS‐R (recorded in the same week). Light blue line is the diagonal, and the red line the linear fit.

**Figure S2:** Adherence of digital assessment
**Note:** Deep blue shading represents high adherence indicating participants conducted this assessment in this study week. Light shading represents low adherence indicating fewer participants conducted the assessment in that study week.

**Figure S3:** Participant feedback per assessment at three visits (negative feedback in gray and positive feedback in blue) at month 1, 5 and 9 (end of study)
**Note:** Scores are scaled from 0 (negative) to 5 (positive). Our original questionnaire had questions with reversed scale as well as questions with only three answers and free text (not included in this figure).

**Figure S4:** Cross‐sectional (A) Clock drawing and fine motor function. Composite score for simple motor function. (B) Speech—speaking rate and bulbar scores. (C) Speech, monotonicity of speech and respiratory function. (D) Spiral drawing and fine motor scores. Mean pressure on pen tip on dominant hand and spiral tracing task. (E) SVC percent predicted volume and respiratory scores. (F) Tongue maximum pressure and bulbar scores. (G) Time Up and Go, total duration and gross motor scores.
Note: Digital metrics for each of the questions and and scores per ALSFRS‐R functional domain with highest absolute regression coefficient (est), *p*‐values (p) and number of samples are displayed in labels. Values are normalized to *z*‐scores.

**Figure S5:** Longitudinal plots. (A) Clock drawing, percent time while pen is not moving. (B) Maximum tongue pressure from IOPI. (C) Speech, speaking rate. (D) Spiral drawing, mean angular velocity on dominant hand during free drawing. (E) Percent predicted volume for hand‐held SVC. (F) Duration of Time Up and Go.

**Data S1:** Description of clinical scales.

**Table S1:** Self‐administered home‐based ALSFRS‐R questionnaire.

**Table S2:** Adherence summary for digital assessments.

**Table S3:** Study participants' feedback.

## Data Availability

The data that support the findings of this study are available from the corresponding author upon reasonable request and Novartis data sharing policies.
