# Supplementary figures and images for: Evaluation of Digital Technologies for Home‐Based Assessment in People With Amyotrophic Lateral Sclerosis
Source: Ann Clin Transl Neurol. 2026 May 20:10.1002/acn3.70429. Online ahead of print. doi: 10.1002/acn3.70429 (PMC13394927; doi:10.1002/acn3.70429)

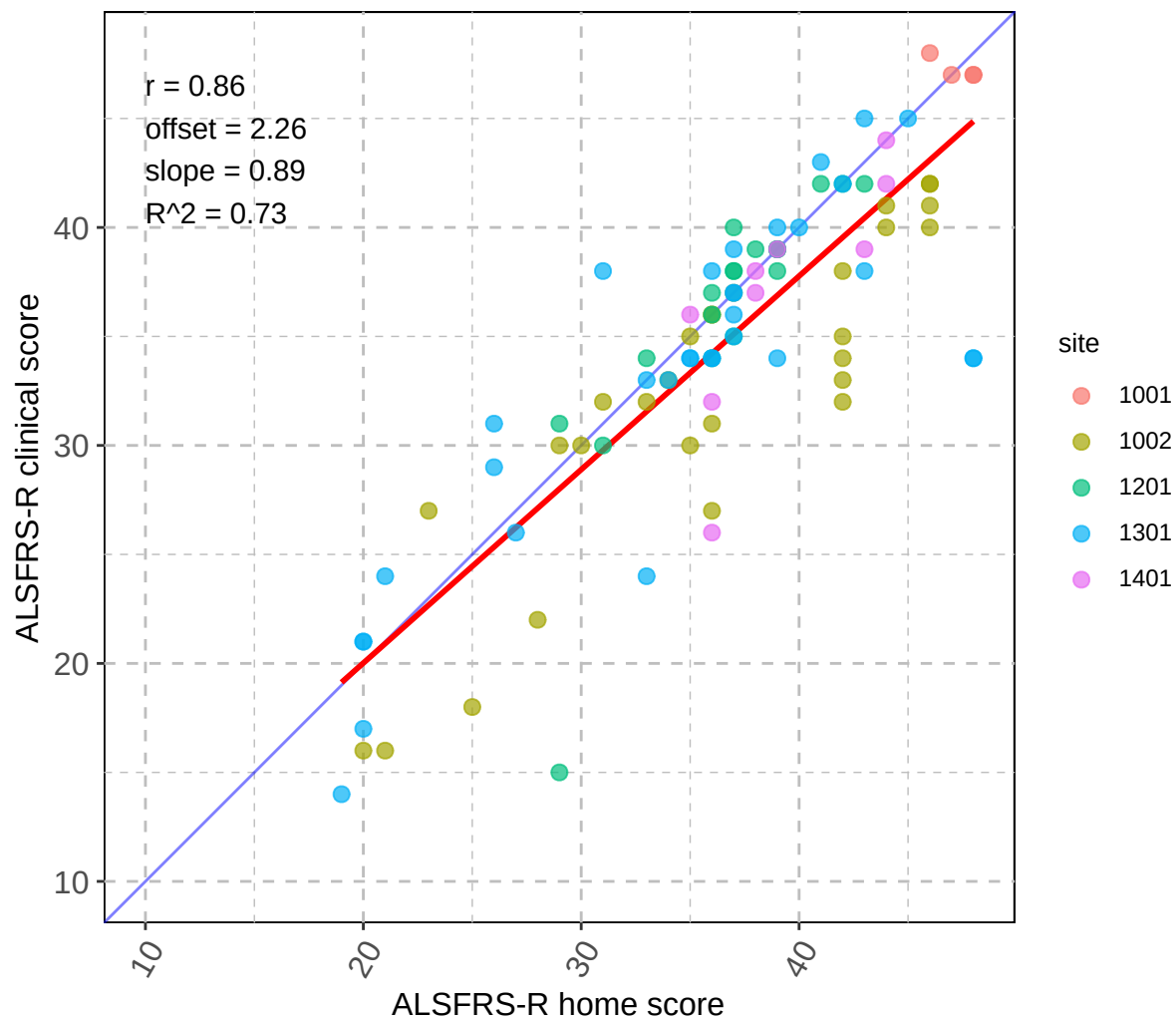

Supplement: Supplementary file 1 — Figure S1: Comparison of self‐administered home and clinic administered ALFRS‐R (recorded in the same week). Light blue line is the diagonal, and the red line the linear fit. [file ACN3-9999-0-s006.pdf]

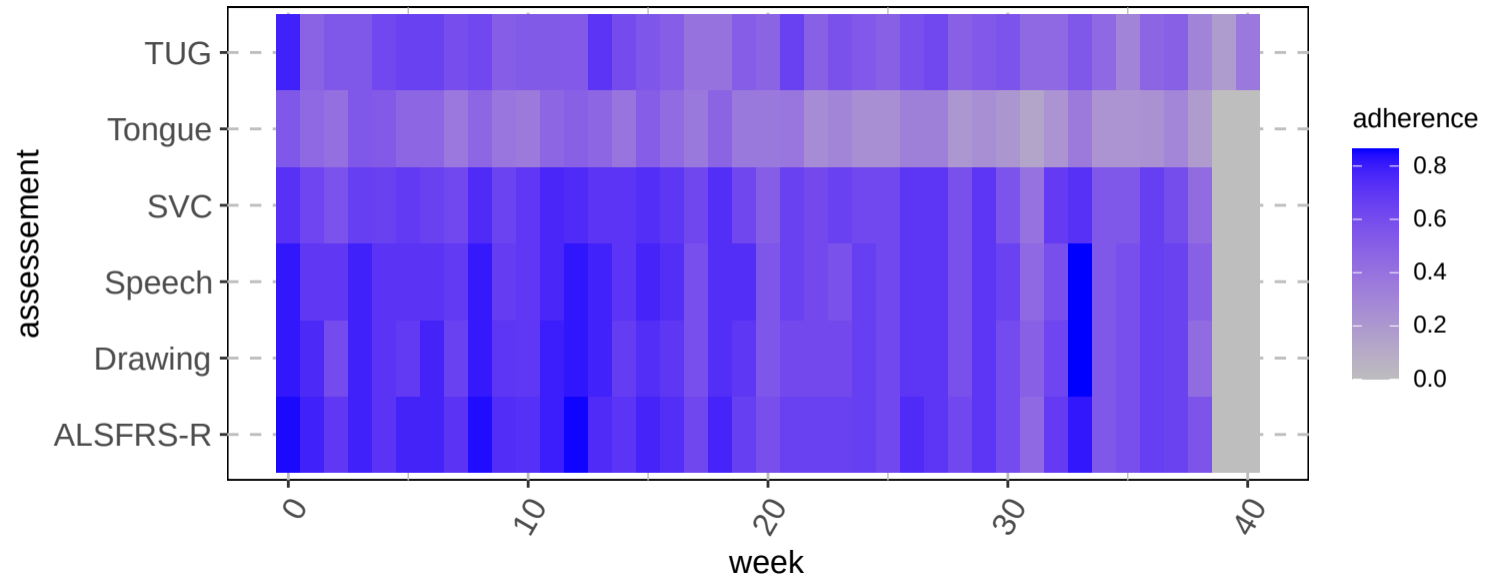

Supplement: Supplementary file 2 — Figure S2: Adherence of digital assessment Note: Deep blue shading represents high adherence indicating participants conducted this assessment in this study week. Light shading represents low adherence indicating fewer participants conducted the assessment in that study week. [file ACN3-9999-0-s009.pdf]

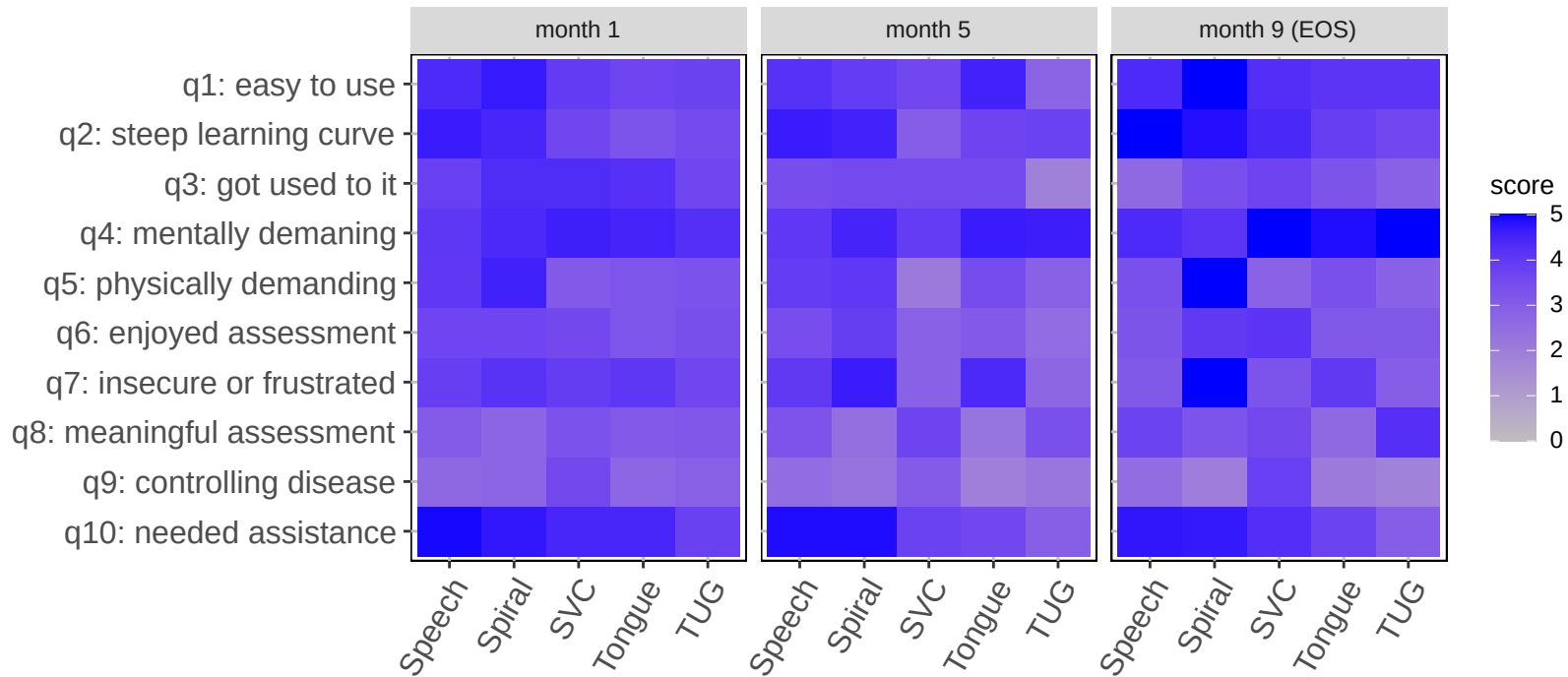

Supplement: Supplementary file 3 — Figure S3: Participant feedback per assessment at three visits (negative feedback in gray and positive feedback in blue) at month 1, 5 and 9 (end of study) Note: Scores are scaled from 0 (negative) to 5 (positive). Our original questionnaire had questions with reversed scale as well as questions with only three answers and free text (not included in this figure). [file ACN3-9999-0-s002.pdf]

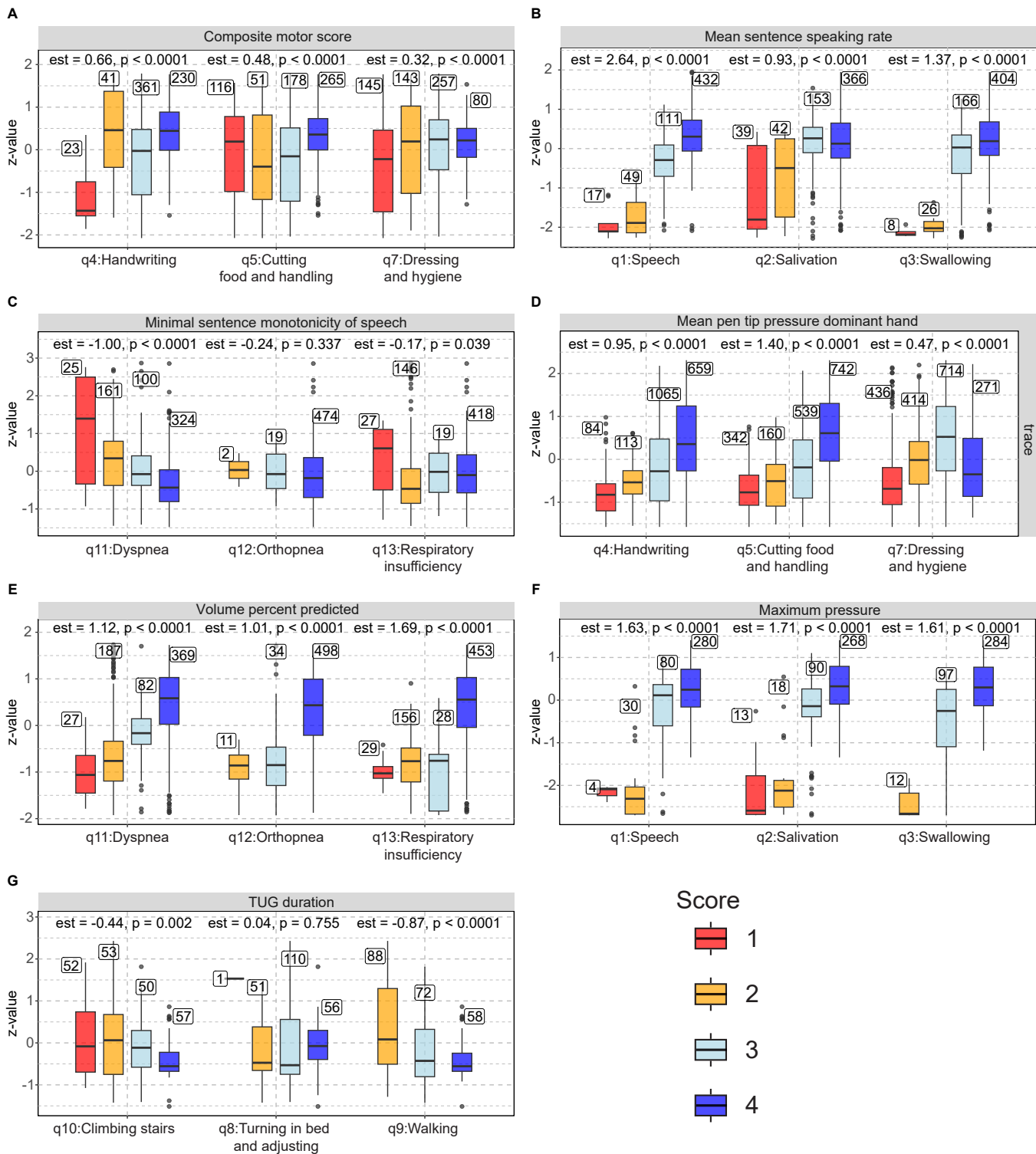

Supplement: Supplementary file 4 — Figure S4: Cross‐sectional (A) Clock drawing and fine motor function. Composite score for simple motor function. (B) Speech—speaking rate and bulbar scores. (C) Speech, monotonicity of speech and respiratory function. (D) Spiral drawing and fine motor scores. Mean pressure on pen tip on dominant hand and spiral tracing task. (E) SVC percent predicted volume and respiratory scores. (F) Tongue maximum pressure and bulbar scores. (G) Time Up and Go, total duration and gross motor scores. Note: Digital metrics for each of the questions and and scores per ALSFRS‐R functional domain with highest absolute regression coefficient (est), p‐values (p) and number of samples are displayed in labels. Values are normalized to z‐scores. [file ACN3-9999-0-s008.pdf]

**A**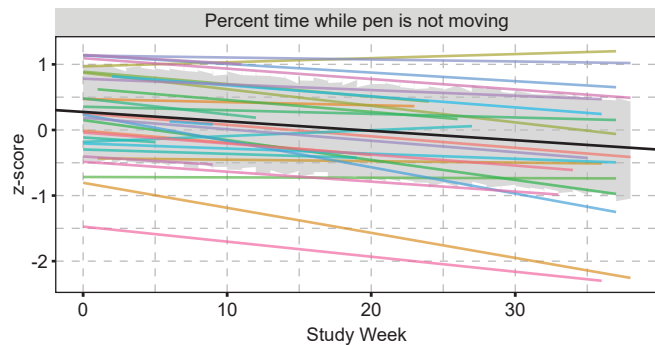**B**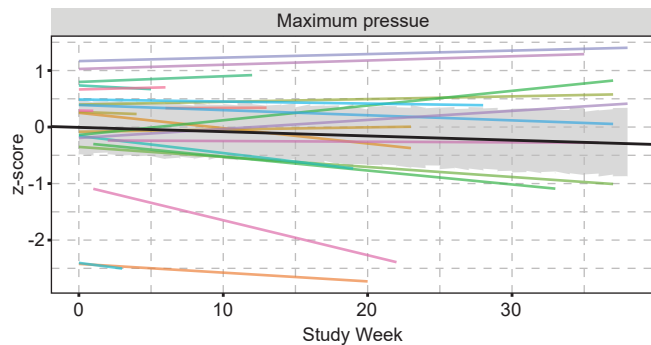**C**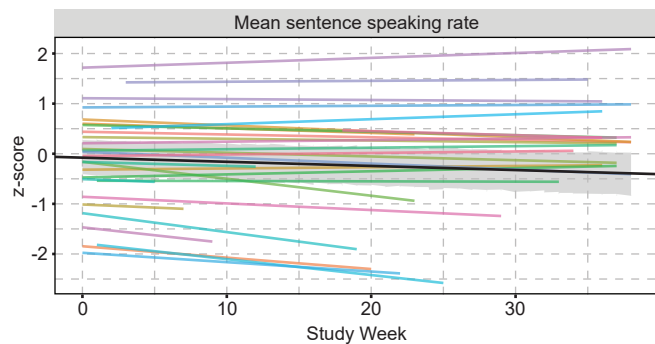**D**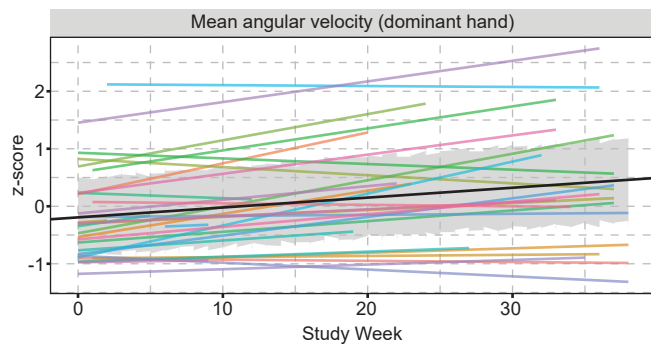**E**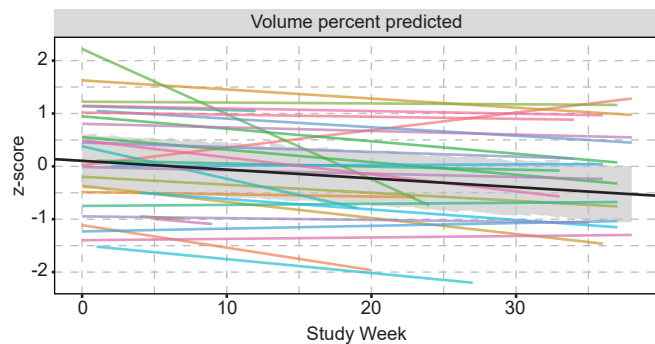**F**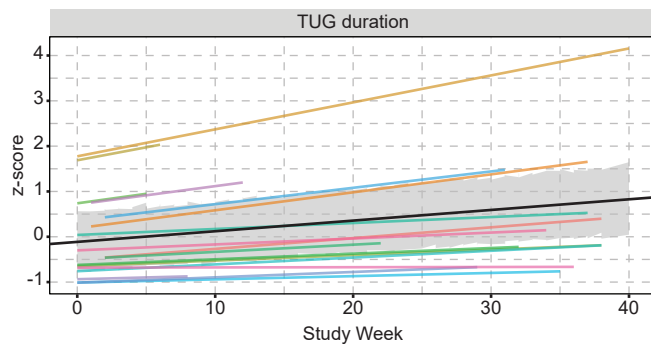

Supplement: Supplementary file 5 — Figure S5: Longitudinal plots. (A) Clock drawing, percent time while pen is not moving. (B) Maximum tongue pressure from IOPI. (C) Speech, speaking rate. (D) Spiral drawing, mean angular velocity on dominant hand during free drawing. (E) Percent predicted volume for hand‐held SVC. (F) Duration of Time Up and Go. [file ACN3-9999-0-s004.pdf]
