# Supplementary material for: Evaluation of Digital Technologies for Home‐Based Assessment in People With Amyotrophic Lateral Sclerosis
Source: Ann Clin Transl Neurol. 2026 May 20:10.1002/acn3.70429. Online ahead of print. doi: 10.1002/acn3.70429 (PMC13394927; doi:10.1002/acn3.70429)
Supplement: Supplementary file 6 — Data S1: Description of clinical scales. [file ACN3-9999-0-s005.docx]

**Supplementary material 1**

**Questionnaires and Scales**

*ALS Functional Rating Scale - Revised (ALSFRS-R)*

ALSFRS-R [1-3] is the standard for assessing functional disability and disease progression in people with ALS. The questionnaire includes 12 questions divided in 4 functional domains : (i) Bulbar function: speech, salivation and swallowing, (ii) Fine motor function: writing, cutting food-using utensils, dressing and hygiene (iii) Gross motor function: turning in bed and adjusting bedclothes, walking and climbing stairs., (iv) Respiratory function: dyspnea, orthopnea, respiratory insufficiency, within each measure, ability to perform the measure is rated from 0 to 4 (total maximum score 48, for normal function). The numbering of the ALSFRS-R in our app splits question 5a and 5b into 5 and 6 (still mutually exclusive) and we therefore have 13 questions.

*Amyotrophic Lateral Sclerosis Assessment Questionnaire (ALSAQ-40)*

ALSAQ-40 [4-6] is a self-reported PRO scale, used specifically to measure the subjective well-being of individuals with ALS. It includes 40 items / questions with 5 discrete sub-scales (each with a maximum 100 for worst condition, e.g. a total maximum of 500): (i) physical mobility (10 items), (ii) activities of daily living and independence (10 items), (iii) eating and drinking (3 items), (iv) communication (7 items), and (v) emotional reactions (10 items). Participants were asked to rate how often a statement is true (e.g., I have found it difficult to feed myself), using a 5 point (Likert scale: never/rarely/sometimes/often/always or cannot do at all.

*EuroQol five-dimensions five-levels (EQ-5D-5L)*

EQ-5D-5L [7] is a self-administered instrument for measuring health-related quality of life. The EQ-5D-5L consists of 2 sections: (i) EQ-5D descriptive system, including five dimensions: mobility, self-care, usual activities, pain/discomfort and anxiety/depression, where the patient indicates his/her health state on 5-level scale from no problems to extreme problems, and (ii) EQ visual analogue scale (EQ VAS) records self-rated health on a vertical visual analogue scale, where the endpoints are labelled ‘The best health you can imagine’ to the ‘The worst health you can imagine’. VAS score with a maximum of 100 for best health.

*McGill Quality of Life scale (McGill QoL)*

The McGill QoL [8] is a self-administered 17-item multidimensional tool (maximum 120 for worst condition) designed to measure physical well-being, physical symptoms, psychological symptoms, existential well-being and support, as well as overall quality of life. We only used part C of the questionnaire to derive a numeric total score (reversing negative questions) with a total of 12 questions ranging from 0 (worst feelings) to 120 (best feelings).

*Zarit caregiver burden interview (ZBI)*

ZBI [9] is used to assess burden among caregivers. Dimensions reported (i.e., 22 items rated on a 5-point Likert scale, maximum 88 for the worst condition) include consequences of caregiving, patient’s dependence, exhaustion and uncertainty, guilt or self-criticism, embarrassment/ anger or frustration, psychological burden and emotional reactions, personal strain, and role strain. The ZBI was completed by the study partner.

*Participant and study partner feedback survey*

The feedback survey includes ten questions per digital endpoint with answers scoring the participant feedback from 0 to 5. Questions cover the following themes: assistance; controlling disease; meaningfulness; insecure or frustrated; enjoyed assessment; physically demanding; mentally demanding; getting used to the assessment; learning curve; ease of use. See **Supplementary Table 3** for details.

**Digital Endpoints**

Digital endpoint assessments included speech/dysarthria, hand fine motor function, tongue strength, slow vital capacity (SVC) and lower extremity function (TUG).

*Speech/Dysarthria*

Participants were asked to read ten sentences, hold the sound “ah” as long possible after a deep breath and repeat the word buttercup as many times as possible in 8 seconds. Speech was recorded with the iPad microphone into an app and analyzed for various metrics such as speaking rate (Aural Analytics, Linus Health) [10].

*Hand fine motor function*

Participants were asked to trace and draw 3 spirals with their dominant hand and 3 spirals with their non-dominant hand each. The test is repeated for tracing a given spiral and free-drawing (no trace) with an apple pen on an iPad. Parameters from a digital clock drawing test (DCT, Linus Health) were analyzed for hand function, even though this test is a cognitive test, its complex drawing may reflect limitations in hand fine motor function.[11,12] Participants drew two clocks: (i) a face of a clock with a specific time and (ii) copy from a face of a clock.

*Tongue strength*

The IOPI® [13] measured the maximum pressure (Pmax) a participant can produce against an air-filled bulb when it is compressed with maximal force by the tongue against the hard palette. The process of uploading data from the IOPI® devices involved connecting them to a computer via USB at the clinical site.

*Slow vital capacity (SVC)*

Using a handheld spirometer and application (ZephyrX) [14], participants performed an SVC test, at least three times per session but not more than 8 times, depending on the performance of a successful trial according to pre-determined acceptable session metrics.

*Timed up and go (TUG)*

Timed “Up & Go” test (TUG) is a quick, reliable and valid test for quantifying functional mobility that may also be useful in following clinical change over time [15]. The TUG measures the time it takes to stand up from a straight-backed chair, walk 3 meters (9.8 feet), turn, walk back to the chair, and sit down. During the performance of the TUG, a sensor with an accelerometer and gyroscope (Physiolog6, MindMaze, Lausanne, Switzerland) was attached on the participant’s hip to measure parameters such as duration, cadence and turning speed during the different sections of the test.

*ALSFRS-R, self-administered*

A unique version of the ALSFRS-R [1-3] for weekly self-administration at home, was developed using simple language that accommodated the character limit on the application and was added as an extra file to the supplementary materials (See **Supplementary Table 1** for details).

**Reference**

1. Cedarbaum JM, Stambler N, Malta E, et al. The ALSFRS-R: a revised ALS functional rating scale that incorporates assessments of respiratory function. BDNF ALS Study Group (Phase III). J Neurol Sci. 1999;169(1-2):13-21. doi: 10.1016/s0022-510x(99)00210-5.
2. Castrillo-Viguera C, Grasso DL, Simpson E, Shefner J, Cudkowicz ME. Clinical significance in the change of decline in ALSFRS-R. Amyotroph Lateral Scler. 2010;11(1-2):178-80. doi: 10.3109/17482960903093710.
3. Montes J, Levy G, Albert S, et al. Development and evaluation of a self-administered version of the ALSFRS-R. Neurology. 2006;67(7):1294-6. doi: 10.1212/01.wnl.0000238505.22066.fc.
4. Jenkinson C, Fitzpatrick R, Brennan C, et al. Development and validation of a short measure of health status for individuals with amyotrophic lateral sclerosis/motor neurone disease: the ALSAQ-40. J Neurol. 1999; 246 Suppl 3:III16-21. doi: 10.1007/BF03161085.
5. Jenkinson C, Fitzpatrick R, Brennan C, Swash M. Evidence for the validity and reliability of the ALS assessment questionnaire: the ALSAQ-40. Amyotroph Lateral Scler Other Motor Neuron Disord. 1999;1(1):33-40. doi: 10.1080/146608299300080022.
6. Jenkinson C, Levvy G, Fitzpatrick R, Garratt A. The amyotrophic lateral sclerosis assessment questionnaire (ALSAQ-40): tests of data quality, score reliability and response rate in a survey of patients. J Neurol Sci. 2000;180(1-2):94-100. doi: 10.1016/s0022-510x(00)00420-2.
7. EuroQol Research Foundation (2019) EQ-5D-5L user guide. In: EuroQol research foundation.
8. Cohen SR, Mount BM, Strobel MG, Bui F. The McGill Quality of Life Questionnaire: a measure of quality of life appropriate for people with advanced disease. A preliminary study of validity and acceptability. Palliat Med. 1995; 9(3):207-19. doi: 10.1177/026921639500900306.
9. Ankri J, Andrieu S, Beaufils B, Grand A, et al. Beyond the global score of the Zarit Burden Interview: useful dimensions for clinicians. Int J Geriatr Psychiatry. 2005; 20(3):254-60. doi: 10.1002/gps.1275.
10. Stegmann GM, Hahn S, Liss J, et al. Early detection and tracking of bulbar changes in ALS via frequent and remote speech analysis. NPJ Digit Med. 2020;3:132. doi: 10.1038/s41746-020-00335-x.
11. Souillard-Mandar W, Penney D, Schaible B, et al. DCTclock: Clinically-Interpretable and Automated Artificial Intelligence Analysis of Drawing Behavior for Capturing Cognition. Front Digit Health. 2021;3:750661. doi: 10.3389/fdgth.2021.750661.
12. Souillard-Mandar W, Davis R, Rudin C, et al. Learning Classification Models of Cognitive Conditions from Subtle Behaviors in the Digital Clock Drawing Test. Mach Learn. 2016;102(3):393-441. doi: 10.1007/s10994-015-5529-5.
13. IOPI Medical. Qualitative Guidelines for Interpreting Tongue Elevation Strength (Pmax). IOPI Normal Values. 2025. Available online: https://iopimedical.com/normal-values/. Accessed on 10 November 2025.
14. ZephyRx. Available online: https://www.zephyrx.com/. Accessed on 10 November 2025.
15. Podsiadlo D, Richardson S. The timed "Up & Go": a test of basic functional mobility for frail elderly persons. J Am Geriatr Soc. 1991;39(2):142-148. doi:10.1111/j.1532-5415.1991.tb01616.x.
