# Supplementary material for: Evaluation of Digital Technologies for Home‐Based Assessment in People With Amyotrophic Lateral Sclerosis
Source: Ann Clin Transl Neurol. 2026 May 20:10.1002/acn3.70429. Online ahead of print. doi: 10.1002/acn3.70429 (PMC13394927; doi:10.1002/acn3.70429)
Supplement: Supplementary file 7 — Table S1: Self‐administered home‐based ALSFRS‐R questionnaire. [file ACN3-9999-0-s007.docx]

**Supplementary Table 1.** Self-administered homa-based ALSFRS-R questionnaire

| **ID** | **Category** | **Question** | **Answer** | **Score** |
| --- | --- | --- | --- | --- |
| q1 | SPEECH | How is your speech? | - Normal - your speech has not changed. | 4 |
|  |  |  | - Detectable speech disturbance - you or your family have noticed a change in your speech. | 3 |
|  |  |  | - Intelligible with repeating - you need to repeat yourself more than 25% of the time for others to understand you. | 2 |
|  |  |  | - Speech combined with non-vocal communication - you use writing or a device to supplement your communication. | 1 |
|  |  |  | - Loss of useful speech - you only communicate by writing or with a communication device. | 0 |
| q2 | SALIVATION | How is your saliva? | - Normal - you do not have any excess saliva. | 4 |
|  |  |  | - Slight but definite excess of saliva - you feel you have excess of saliva, but you do not need a tissue. You may also have nighttime drooling. | 3 |
|  |  |  | - Moderately excessive saliva - you use a tissue less than 25% of the time to manage saliva. | 2 |
|  |  |  | - Marked excess of saliva - you have some drooling and often use a tissue but not always. | 1 |
|  |  |  | - Marked drooling - you require the constant use of tissue or handkerchief, or suction. | 0 |
| q3 | SWALLOWING | How is your swallowing? | - Normal - there is no change in your swallowing. | 4 |
|  |  |  | - Early eating problems - occasionally food will stick and cause coughing or choking. Food may need to be cut up small. | 3 |
|  |  |  | - Dietary consistency changes - food needs to be mashed or liquidized, drinks may need thickener, or some foods need to be avoided. | 2 |
|  |  |  | - Needs supplemental tube feeding - oral intake of food is so difficult you use a feeding tube to supplement your caloric intake | 1 |
|  |  |  | - NPO - you receive all of your nutrition through a feeding tube. | 0 |
| q4 | HANDWRITING | How is your handwriting? | - Normal - your handwriting has not changed. | 4 |
|  |  |  | - Slow or sloppy, all words are legible - there is a change in handwriting, but all the words are legible. | 3 |
|  |  |  | - Not all words are legible - some of the words you write cannot be read but other words can. | 2 |
|  |  |  | - No words are legible, but can still grip pen - you can still hold a pen, but you cannot write. | 1 |
|  |  |  | - Unable to grip pen - you are not able to hold a pen in your hand. | 0 |
| q5 | CUTTING FOOD AND HANDLING UTENSILS: | How are you with cutting food or handling cutlery? | - Not Applicable - If you use a feed tube for nutritional intake, skip to the next question. | NA |
|  |  |  | - Normal - there is no change in your ability to cut food or handle a utensil. | 4 |
|  |  |  | - Somewhat slow and clumsy, but no help needed - there is some difficulty, but you are still able to do this independently. | 3 |
|  |  |  | - Can cut most foods although slow and clumsy; some help needed - you occasionally need assistance. | 2 |
|  |  |  | - Food must be cut by someone but can still feed slowly - you need assistance at least half the time for cutting food but not for feeding. | 1 |
|  |  |  | - Needs to be fed - someone must feed you because you are not able to cut food or handle a utensil. | 0 |
| q6 | CUTTING FOOD AND HANDLING UTENSILS: | How are you with handling the gastrostomy fastenings and fixtures? | - Not applicable if you eat food by mouth and do not use a feeding tube for nutritional intake. | NA |
|  |  |  | - Normal - you have no difficulty at all with any of the manipulations. | 4 |
|  |  |  | - Clumsy, but able to perform all manipulations independently - you have some difficulty but you are able to perform tube feedings independently | 3 |
|  |  |  | - Some help needed with closures and fasteners - you need someone to assist you with the tube feeding manipulations. | 2 |
|  |  |  | - Provides minimal assistance to caregiver - you are able to provide some assistance but need a caregiver to perform most of the task. | 1 |
|  |  |  | - Unable to perform any aspect of task - you are not able to assist with any part of the task. | 0 |
| q7 | DRESSING AND HYGIENE | How are you doing with dressing or washing? | - Normal - there is no change in your ability to dress or wash yourself. | 4 |
|  |  |  | - Independent but with effort or decreased efficiency - you are slower than before, but do not need assistance from another person or device. | 3 |
|  |  |  | - Intermittent assistance or substitute methods - you are generally independent but may need help from a caregiver or devices. | 2 |
|  |  |  | - Needs attendant for self-care - you need assistance with all aspects dressing or washing, but you are able to assist the caregiver. | 1 |
|  |  |  | - Total dependence - you cannot help your caregiver with dressing or washing | 0 |
| q8 | TURNING IN BED AND ADJUSTING BED CLOTHES | How are you with turning in bed and adjusting the sheets and blankets? | - Normal - there is no change in your ability to turn in bed or adjust the covers. | 4 |
|  |  |  | - Somewhat slow and clumsy, but no help needed - you have some difficulty with either turning in bed or adjusting the sheets and blankets. | 3 |
|  |  |  | - Can turn alone, or adjust sheets, but with great difficulty - you can either turn in bed or adjust the sheets and blankets but it is very difficult. | 2 |
|  |  |  | - Can initiate, but not turn or adjust sheets alone - you can start turning or adjusting the covers but need assistance in order to complete the task. | 1 |
|  |  |  | - Helpless - that you are not able to initiate turning or adjust the covers. | 0 |
| q9 | WALKING | How is your walking? | - Normal - there is no change in your walking ability. | 4 |
|  |  |  | - Early ambulation difficulties - you might have some difficulty walking or walk more slowly, but you do not need assistance. | 3 |
|  |  |  | - Walks with assistance - you need assistance from a device or from a caregiver in order to walk. | 2 |
|  |  |  | - Non-ambulatory functional movement - you can move your legs and stand up, but cannot walk. | 1 |
|  |  |  | - No purposeful leg movement - you cannot move your legs. | 0 |
| q10 | CLIMBING STAIRS | How are you doing with climbing stairs? | - Normal - there is no change in your ability to go up stairs. | 4 |
|  |  |  | - Slow - it takes more time to go upstairs, but you do not feel unsteady or need to rest between steps. | 3 |
|  |  |  | - Mild unsteadiness or fatigue - you need to rest or feel unsteady when going up stairs. | 2 |
|  |  |  | - Needs assistance - you have to use the handrail, or the help from a caregiver to climb stairs. | 1 |
|  |  |  | - Cannot do - you are not able to climb stairs. | 0 |
| q11 | DYSPNEA | Do you have shortness of breath? | - None - you do not experience shortness of breath. | 4 |
|  |  |  | - Occurs when walking - you experience shortness of breath when you are walking. | 3 |
|  |  |  | - Occurs with one or more of the following: eating, bathing, dressing - you experience shortness of breath when you are eating or bathing or dressing. | 2 |
|  |  |  | - Occurs at rest - you have difficulty breathing when you are sitting or lying down to rest. | 1 |
|  |  |  | - Significant difficulty - you consistently experience shortness of breath and are considering using some type of breathing assistance. | 0 |
| q12 | ORTHOPNEA | Orthopnea is shortness of breath or difficulty breathing when lying flat. Do you have any difficulty breathing when lying flat? | - None - you do not have any shortness of breath or difficulty breathing when lying flat. | 4 |
|  |  |  | - Some difficulty sleeping at night due to shortness of breath - you may have difficulty sleeping due to shortness of breath but do not use more than 2 pillows to sleep. | 3 |
|  |  |  | - Needs extra pillows in order to sleep - you need more than 2 pillows in order to sleep without shortness of breath. | 2 |
|  |  |  | - Can only sleep sitting up - you can only sleep sitting up due to shortness of breath. | 1 |
|  |  |  | - Unable to sleep without mechanical assistance - you require the use of breathing support such as BiPAP to sleep and do not sleep without it. | 0 |
| q13 | RESPIRATORY INSUFFICIENCY | Do you require breathing support? | - None - you do not use any breathing support such as BiPAP or a Ventilator. | 4 |
|  |  |  | - Intermittent use of BiPAP - you sometimes use breathing support such as BiPAP during the day or during the night. | 3 |
|  |  |  | - Continuous use of BiPAP during the night - you use breathing support such as BiPAP all night long. | 2 |
|  |  |  | - Continuous use of BiPAP during the day and the night - you use breathing support such as BiPAP during the day and at night. | 1 |
|  |  |  | - Invasive mechanical ventilation - you require use of a ventilator by intubation or tracheostomy at all times. | 0 |

Column 1: a generic id for the question

Column 2: category/functional domain for the question

Column 3: plain text question

Column 4: plain text answer

Column 5: numeric score
