# Supplementary material for: Evaluation of Digital Technologies for Home‐Based Assessment in People With Amyotrophic Lateral Sclerosis
Source: Ann Clin Transl Neurol. 2026 May 20:10.1002/acn3.70429. Online ahead of print. doi: 10.1002/acn3.70429 (PMC13394927; doi:10.1002/acn3.70429)
Supplement: Supplementary file 8 — Table S2: Adherence summary for digital assessments. [file ACN3-9999-0-s003.docx]

**Supplementary Table 2:** Adherence summary for digital assessments

| Assessment | Mean | SD |
| --- | --- | --- |
| ALSFRS-R | 0.67 | 0.18 |
| Drawing | 0.65 | 0.17 |
| SVC | 0.62 | 0.16 |
| Speech | 0.65 | 0.17 |
| TUG | 0.52 | 0.11 |
| Tongue | 0.34 | 0.14 |
