# Supplementary material for: Evaluation of Digital Technologies for Home‐Based Assessment in People With Amyotrophic Lateral Sclerosis
Source: Ann Clin Transl Neurol. 2026 May 20:10.1002/acn3.70429. Online ahead of print. doi: 10.1002/acn3.70429 (PMC13394927; doi:10.1002/acn3.70429)
Supplement: Supplementary file 9 — Table S3: Study participants' feedback. [file ACN3-9999-0-s001.docx]

**Supplementary Table 3.** Study participants’ feedback

|  | **Technology Feasibility – User Friendliness** | | | | | |
| --- | --- | --- | --- | --- | --- | --- |
|  | The device was easy to use. | | | | | |
|  | ⬜ 0 (Strongly disagree) | ⬜ 1 | ⬜ 2 | ⬜ 3 | ⬜ 4 | ⬜ 5 (Strongly agree) |
|  | I needed to learn many things before I could get going with this device. | | | | | |
|  | ⬜ 0 (Strongly disagree) | ⬜ 1 | ⬜ 2 | ⬜ 3 | ⬜ 4 | ⬜ 5 (Strongly agree) |
|  | Since the last visit/survey, I have become more confident in being able to conduct this self-assessment | | | | | |
|  | ⬜ 0 (Strongly disagree) | ⬜ 1 | ⬜ 2 | ⬜ 3 | ⬜ 4 | ⬜ 5 (Strongly agree) |
|  |  | | | | | |
|  | **Mental and physical demand of the task itself** | | | | | |
|  | This assessment was mentally demanding. | | | | | |
|  | ⬜ 0 (Strongly disagree) | ⬜ 1 | ⬜ 2 | ⬜ 3 | ⬜ 4 | ⬜ 5 (Strongly agree) |
|  | This assessment was physically demanding. | | | | | |
|  | ⬜ 0 (Strongly disagree) | ⬜ 1 | ⬜ 2 | ⬜ 3 | ⬜ 4 | ⬜ 5 (Strongly agree) |
|  |  | | | | | |
|  | **Enjoyment and frustration** | | | | | |
|  | I enjoyed this assessment. | | | | | |
|  | ⬜ 0 (Strongly disagree) | ⬜ 1 | ⬜ 2 | ⬜ 3 | ⬜ 4 | ⬜ 5 (Strongly agree) |
|  | I was insecure and/or frustrated during this assessment. | | | | | |
|  | ⬜ 0 (Strongly disagree) | ⬜ 1 | ⬜ 2 | ⬜ 3 | ⬜ 4 | ⬜ 5 (Strongly agree) |
|  |  | | | | | |
|  | **Valuable assessment** | | | | | |
|  | This assessment felt meaningful and relevant to the difficulties I have in my daily life. | | | | | |
|  | ⬜ 0 (Strongly disagree) | ⬜ 1 | ⬜ 2 | ⬜ 3 | ⬜ 4 | ⬜ 5 (Strongly agree) |
|  | Taking my measurement has made me feel more in control of my disease | | | | | |
|  | ⬜ 0 (Strongly disagree) | ⬜ 1 | ⬜ 2 | ⬜ 3 | ⬜ 4 | ⬜ 5 (Strongly agree) |
|  |  | | | | | |
|  | **Study partner support** | | | | | |
|  | Did you need help from study partner to perform the assessments? | | | | | |
|  | ⬜ Never | | ⬜ Sometimes | | ⬜ Always | |
|  | If yes, study partner feedback for this device: | | | | | |
|  | _______________________________________________________________________________________________ | | | | | |
|  | _______________________________________________________________________________________________ | | | | | |
|  | _______________________________________________________________________________________________ | | | | | |
|  |  | | | | | |
|  | **Open comments (study participant)** | | | | | |
|  | What did you like best about this digital device? | | | | | |
|  | _______________________________________________________________________________________________ | | | | | |
|  | _______________________________________________________________________________________________ | | | | | |
|  | _______________________________________________________________________________________________ | | | | | |
|  |  | | | | | |
|  | What did you like least about this digital device? | | | | | |
|  | _______________________________________________________________________________________________ | | | | | |
|  | _______________________________________________________________________________________________ | | | | | |
|  | _______________________________________________________________________________________________ | | | | | |
|  |  | | | | | |
|  | Is there anything else you would like to add? | | | | | |
|  | _______________________________________________________________________________________________ | | | | | |
|  | _______________________________________________________________________________________________ | | | | | |
|  | _______________________________________________________________________________________________ | | | | | |
|  |  | | | | | |

| **Question ID** | **Domain** | **Question** | **Short label** | **Format** | **Worst score** |
| --- | --- | --- | --- | --- | --- |
| q1 | Technology feasibility user friendliness | The device was easy to use. | q1: easy to use | 0 to 5 | 0 |
| q2 | Technology feasibility user friendliness | I needed to learn many things before I could get going with this device. | q2: steep learning curve | 0 to 5 | 5 |
| q3 | Technology feasibility user friendliness | Since the last visit/survey, I have become more confident in being able to conduct this self-assessment | q3: got used to it | 0 to 5 | 0 |
| q4 | Mental and physical demand of the task itself | This assessment was mentally demanding. | q4: mentally demanding | 0 to 5 | 5 |
| q5 | Mental and physical demand of the task itself | This assessment was physically demanding. | q5: physically demanding | 0 to 5 | 5 |
| q6 | Enjoyment and frustration | I enjoyed this assessment. | q6: enjoyed assessment | 0 to 5 | 0 |
| q7 | Enjoyment and frustration | I was insecure and/or frustrated during this assessment. | q7: insecure or frustrated | 0 to 5 | 5 |
| q8 | Valuable assessment | This assessment felt meaningful and relevant to the difficulties I have in my daily life. | q8: meaningful assessment | 0 to 5 | 0 |
| q9 | Valuable assessment | Taking my measurement has made me feel more in control of my disease. | q9: controlling disease | 0 to 5 | 0 |
| q10 | Study partner support | Did you need help from study partner to perform the assessments? | q10: needed assistance | 0 to 2 | 5 |
| q11 | Study partner support | Did you need help from study partner to perform the assessments? | q11: partner feedback | free text | NA |
| q12 | Open comments (study participant) | What did you like best about this digital device? | q12: like best about it | free text | NA |
| q13 | Open comments (study participant) | What did you like least about this digital device? | q13: like least about it | free text | NA |
| q14 | Open comments (study participant) | Is there anything else you would like to add? | q14: comments | free text | NA |
